# Feature fusion network based on strip pooling

**DOI:** 10.1038/s41598-021-00585-z

**Published:** 2021-10-28

**Authors:** Gaihua Wang, Qianyu Zhai

**Affiliations:** 1 School of Electrical and Electronic Engineering, Hubei University of Technology, Wuhan, 430068 China; 2 Hubei Key Laboratory for High-Efficiency Utilization of Solar Energy and Operation Control of Energy Storage System, Hubei University of Technology, Wuhan, 430068 China

**Keywords:** Computer science, Information technology, Software

## Abstract

Contextual information is a key factor affecting semantic segmentation. Recently, many methods have tried to use the self-attention mechanism to capture more contextual information. However, these methods with self-attention mechanism need a huge computation. In order to solve this problem, a novel self-attention network, called FFANet, is designed to efficiently capture contextual information, which reduces the amount of calculation through strip pooling and linear layers. It proposes the feature fusion (FF) module to calculate the affinity matrix. The affinity matrix can capture the relationship between pixels. Then we multiply the affinity matrix with the feature map, which can selectively increase the weight of the region of interest. Extensive experiments on the public datasets (PASCAL VOC2012, CityScapes) and remote sensing dataset (DLRSD) have been conducted and achieved Mean Iou score 74.5%, 70.3%, and 63.9% respectively. Compared with the current typical algorithms, the proposed method has achieved excellent performance.

## Introduction

Semantic segmentation, which is the fundamental and challenging problem in computer vision, is to parse the category of each pixel in the image. It has been extensively researched in a variety of fields, such as autonomous driving, remote sensing images, medical diagnosis, and so on.

With the emergence of Fully Convolutional Neural Networks (FCNs)^1^, many methods have made remarkable progress in semantic segmentation. However, due to the limitations of the network structure, the traditional FCN only obtains the local information of the image and lacks sufficient contextual information, which can easily lead to incorrect segmentation results.

In recent years, many novel networks^2–7^ have tried to seek new methods to solve FCN's issues. UperNet^8^ uses feature pyramid network (FPN) to capture multi-scale features and analyze different scenes. DenseASPP^9^ combines dense connection with ASPP, which is composed of the dilated convolution with different rates, to generate different receptive fields. Affinity Loss^10^ was proposed by Yu et al. to distinguish the relationship between different pixels. HRNet^11^ maintains high-resolution representations by connecting high-resolution to low-resolution convolutions in parallel. LedNet^12^ uses attention pyramid network (APN) to capture contextual information, and uses convolutional decomposition and channel separation to reduce network complexity. HANet^13^ introduces a highly-driven attention module to improve image segmentation in urban scenes. SPNet^14^ proposes the strip pooling to solve the long-term dependence of the network.

In order to complete the semantic segmentation task more quickly and accurately, a novel semantic segmentation network is designed, which can efficiently aggregate context information. Specifically, it consists of a series of convolution branches and two FF modules. The FF module uses strip pooling and two linear layers to generate the affinity matrix, which can capture the correlation between any features. For each spatial position on the affinity matrix, it collects all the information from the local feature map. The main contributions of this study can be summarized as follows:

1. We design a new network with self-attention mechanism, to solve the long-term dependence problem in semantic segmentation tasks.

2. An FF module is proposed to reduce the computational cost of affinity matrix. It efficiently captures contextual information by converting matrix multiplication to vector multiplication.

3. The experiments show that the proposed method has better performance on three mainstream benchmarks including PASCAL VCO 2012, Cityscapes and DLRSD.

The remaining paper is organized in the following way. “Related work” examines the top-ranking related work on semantic segmentation. The proposed method is introduced in “Methods”. In “Experiments”, We have conducted a large number of ablation and comparative experiments to verify the effectiveness of the proposed method. “Conclusion” is the summary of this paper.

## Related work

### Context information

In semantic segmentation, contextual information aggregation can significantly improve the segmentation effect. Some methods with aggregating context information have been studied. DeepLabs^15,16^ uses dilated convolutions with different rates to stack the ASPP (atrous spatial pyramid pooling) structure to capture different contextual information. Zhao et al.^17^ stacked pooling layers of different sizes to form a pyramid structure to capture more contextual information. GCN^18^ aggregates more context information by using a larger convolution kernel and reduces the number of parameters by convolution decomposition. Some works, such as SegNet^19^, U-Net^20^, U-Net++^21^ and ENet^22^, use encoding–decoding structures to aggregate low-dimensional information. In addition, the attention mechanism is also introduced to aggregate context information. DFNet^23^ and EncNet^24^ are inspired by SENet^25^ and assign different weights to different channels. Zhao et al.^26^ proposed a PSA module to generate an over-completed map to adaptively aggregate context information. These methods with aggregating certain contextual information can assist the network focus on areas of interest effectively.

### Self-attention mechanism

Long-term dependence is a significant issue that influences the effect of semantic segmentation. One solution is to use the self-attention mechanism^27^ which is first applied in the field of natural language processing to calculate the affinity matrix between each pixel. It can be expressed as:

1 $$Attenion(Q,K,V)=\mathit{soft \,\,max}(\frac{Q{K}^{T}}{\sqrt{dk}})V$$

Q, K, and V are weight matrixes, and $\sqrt{dk}$ is a constant. The self-attention mechanism needs to multiply two matrices, which will cause huge computation.

The non-local^28^ module employs the relationship between two locations to capture long-term dependence of the network, which enable each position on the feature map to obtain information of other positions. CCNet^29^ has designed a CC module to capture the horizontal and vertical direction information. By repeatedly stacking the CC modules, the similarity of any two positions on the feature map can be calculated. DANet^30^ and DRANet^31^ learn the correlation between space and channel respectively through position and channel attention modules. TANet^32^ combines channel and spatial attention to improve the segmentation effect. OCNet^33^ combines the self-attention mechanism with the ASPP structure.

Different from previous self-attention modules, we design a lightweight self-attention module based on strip pooling, which can capture global information more efficiently and uses the linear layer to make up for the loss of information caused by the pooling operation.The proposed self-attention module transforms the original matrix multiplication into vector multiplication, which greatly reduces the cost of computation and memory.

## Method

This section first introduces the overall architecture of the network and describes the FF module that can capture sparse local information. Then recurrent the FF module to capture the dense global information. Finally it describes how to aggregate all the module together.

### Network architecture

**Figure 1:**
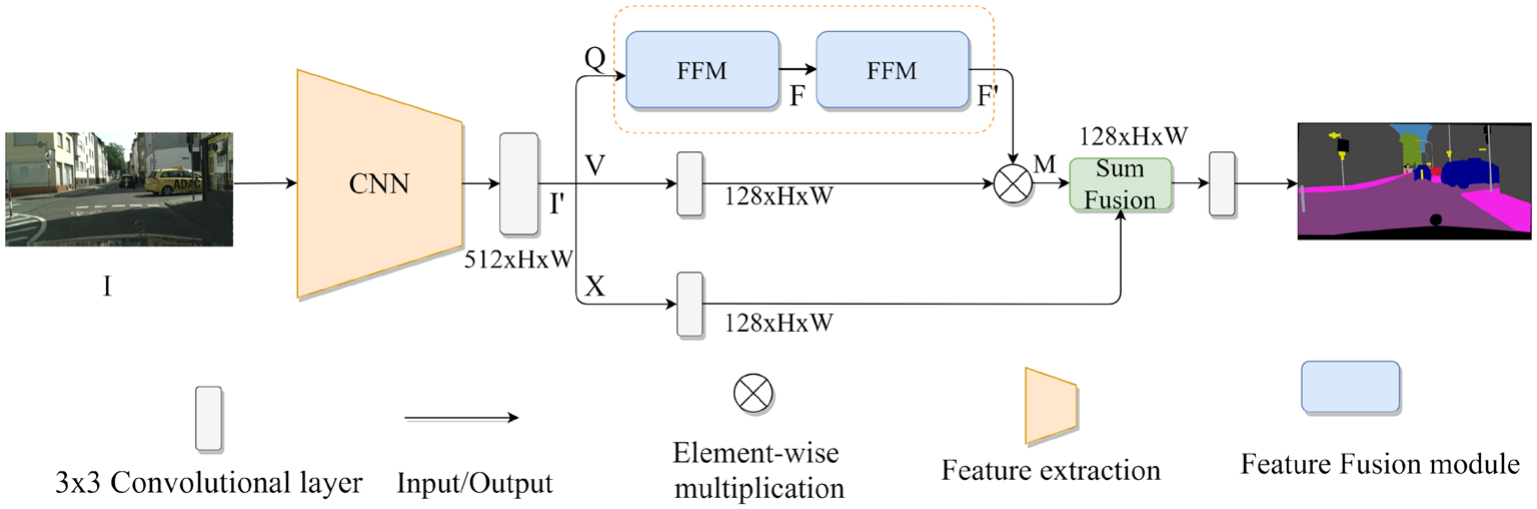
The overall structure of our network.

The overall architecture of the network is shown in Fig. 1. The convolutional layer in the figure represents a convolution, BN, and ReLU. CNN uses ResNet50 with dilated convolution. To retain more detailed information, dilated convolution is used in the last two blocks of ResNet50. The height and width of the output are 1/8 of the input I. The extracted feature is processed by 3 × 3 convolutional layer to get I' (the number of channels decreased from 2048 to 512). Then, the network with Q, V, and X branches is designed. The Q has two serial FF modules. The first FF module generates feature map F by extracting information in the horizontal and vertical directions. The second FF module generates the affinity matrix F', which is the result of the weighted summation of all pixels. The V and X directly reduce the channel dimension through the 3 × 3 convolution with BN and ReLU (the number of channels decreased from 512 to 128). And the result of V branch and F' are multiplied to generate the attention matrix M. The result of X branchand M are added to enhance the feature representation. Finally, the fused feature map is sent to the convolutional layer and generates ultimate prediction images.

### Feature fusion module

**Figure 2:**
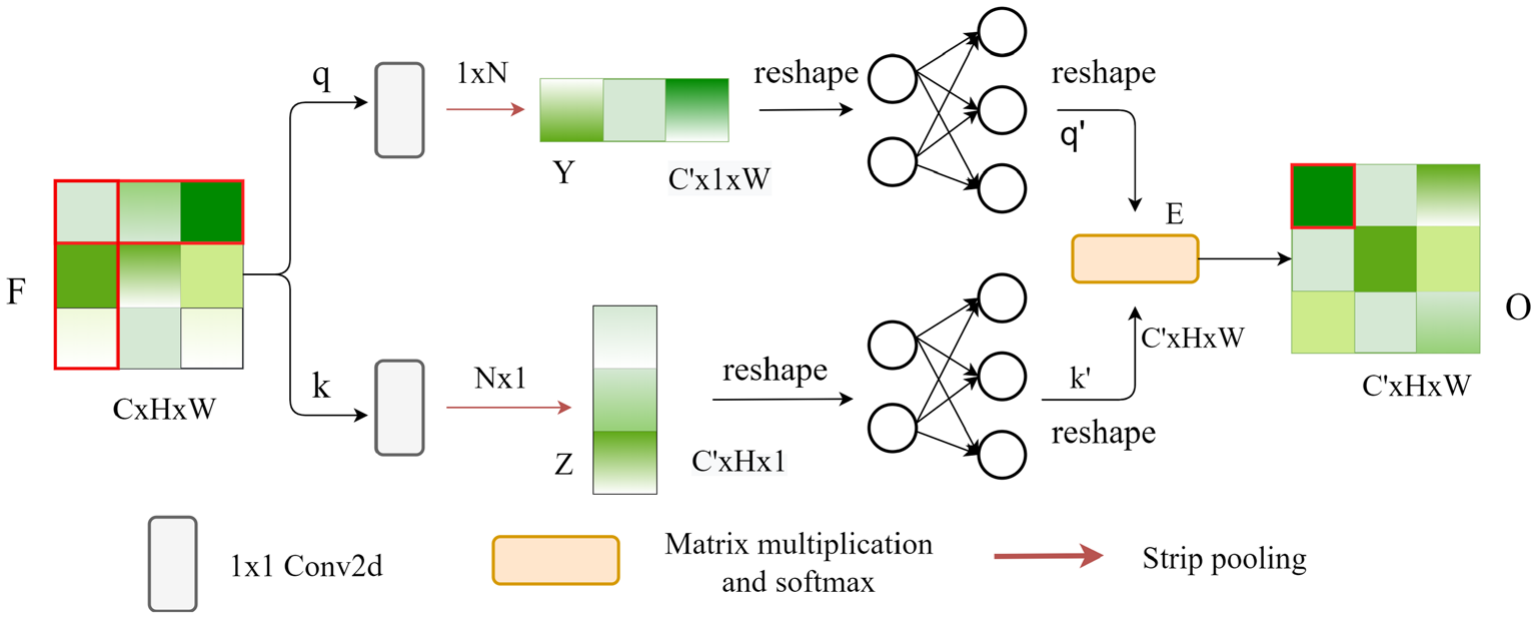
The main architecture of the FF module.

As shown in Fig. 2, given feature map *F* (CxHxW), which divides into two branches q and k. In the q branch, it performs 1 × 1 convolutional to reduce dimension to C' × H × W (C' is half of the C) and a column average pooling to compress height dimension to get *Y* (C' × 1 × W), where *Y* ∈ R. Then reshapes to C' × W (remove height dimension) and gets feature vector q' by using two linear layers. Among them, the function of the linear layer is to convert the strip pooling result and reduce feature loss caused by strip pooling. It is worth noting that the output size of the linear layer is C' → C'/4 → C', and they all use linear activation functions. This process can be described as:

2 $$Y = \frac{1}{H}\sum\nolimits_{i = 0}^{H} {t_{i} }$$

3 $$q^{\prime} = g(Y,W)$$

Equation (2) shows the process of column average pooling, where *H* represents the height of the feature map, t_i_ represents the ith element in each column. *Y* is the result of column average pooling. Eq. (3) shows the process of fully connected layer, where *g* represents linear layer*, W* is the learnable weight matrix of the linear layer. It can be found that q' is generated after the input feature map is compressed and then space transformation is performed.

The k branch is similar to the q branch, and k' will be obtained after row average pooling and two linear layers. After reshaping q' and k', matrix multiplication is performed to produce the output *E*. Then, use *E* to generate output *O*. Please note that *O* is equal to *E* in the first FF module, but in the second FF module, *O* is obtained after *E* passes through the Softmax function. As shown in Fig. 2, the way of the FF module collects information is marked in red. Each position on *O* combines information from row and column of the feature map *F*. An FF module cannot collect enough global information, so we feed *O* to the *FF* module again to capture global information, and calculate the affinity matrix between pixels through the Softmax function. Note that the linear layer can only output a fixed size. So,we use 1 × 1 convolution instead of the linear layer to achieve an output of any size. Experiments have also proved that the 1 × 1 convolution and linear layer are equivalent.

## Experiments

we first introduce PASCAL VOC2012, Cityscapes, and DLRSD, then introduce the experimental environment and details, and finally compare and verify the proposed method on different datasets.

### Datasets

PASCAL VOC2012 is a segmentation dataset. It has 21 categories, including airplanes, bicycles, boats, etc. The dataset has 10,582 images for training and 1449 images for verification.

CityScapes is a city segmentation dataset. It collects road landscape images of 50 cities, each image size is 2048 × 1024. The dataset contains 19 common categories in road scenes, with a total of 5000 high-quality pixel-level labels. The training set contains 2979 images, the validation set contains 500 images, and the test set contains 1525 images.

DLRSD is a dense labeling dataset that builds for remote sensing image segmentation tasks. It contains 2100 images with a pixel size of 256 × 256, covering 17 common remote sensing image scene categories. We divide the training set and validation set according to the ratio of 0.8:0.2 for each category.

### Experimental settings

The implementation of our network is based on the Pytorch framework. Its version is 1.1.0, and the CUDA version is 10.0. We only use a Nvidia GTX 1080TI to complete the experiment. Like the previous method, it uses the ‘Poly’ strategy to update the learning rate. The decoder initial learning rates of the PASCAL VOC 2012, DLRSD, and CityScapes datasets are 0.05, 0.008, and 0.01. During the training process, the learning rate of the encoder is 1/10 of that of the decoder. We employ the SGD optimizer, where weight decay and momentum are 0.0001 and 0.9 respectively.

For the backbone network, it chooses ResNet50^34^ with dilated convolution, which has been pre-trained on ImageNet. For the sake of generality, all the networks in the experiment do not use auxiliary loss functions. We use flip, rotate, zoom, random scramble, random crop, and blur operations on the dataset to augment the data. The batch sizes of the PASCAL VOC 2012, and DLRSD are 8, the CityScapes is 4. And the size of the input is randomly cropped to 384, 224, and 512 respectively. The number of epoch of the PASCAL VOC 2012 and DLRSD is 180 and the CityScapes is 120. Besides, the pixel accuracy (PA) and the mean intersection of union (mIoU) are used as the main evaluation indicators of the experiment, and their definitions are as follows:

4 $$mIoU = \frac{1}{k + 1}\sum\nolimits_{i = 0}^{K} {\frac{{{\text{TP}}}}{FN + FP + TP}}$$

5 $$PA = \frac{TP + TN}{{TP + TN + FP + FN}}$$

where *TP* represents true positive, *TN* represents true negative, *FP* represents false positive, *FN* represents false negative, and k represents the number of categories.

## Results analysis

### Ablation study

We use the same hyperparameters for experiments. As shown in Table 1, the ablation experiments on the PASCAL VOC 2012 are performed.

**Table 1:** Results of ablation experiments.

Method	Backbone	FF	mIoU (%)	PA (%)
FCN8s^1^	**ResNet50**^32^	–	64.4	90.8
Ours	**ResNet50**^32^	× 1	72.8	92.9
Ours	MobileNet v2^34^	× 2	67.7	91.2
Ours	EfficientNet b0^35^	× 2	70.4	90
Ours	**ResNet50**^32^	× 2	74.5	93.5
Ours	ResNet101^33^	× 2	75.8	93.8

In the Table1, the second row is the result of one FF module. And the fifth row is the result of two FF modules. Obviously, The FF module can significantly improve the segmentation accuracy. Compared with the base-line FCN8s (use ResNet50 as the backbone network), using an FF module can bring an 8.4% improvement on mIoU. When stacking two FF modules repeatedly, the proposed method can increase mIoU from 72.8% to 74.5%. And it can help the network better aggregate contextual information. We add the FF module to different backbone networks to verify its effectiveness. Like ResNet50, we replace the last convolutional layer of the backbone network with dilated convolutional and fine-tune it. When the FF module is combined with the lightweight backbones MobileNet v2 and EfficientNet b0, 67.7% and 70.4% mIoU can be achieved respectively. It is worth noting that when we use ResNet101, its feature extraction ability is stronger, which can bring the highest mIoU of 75.8%.

**Figure 3:**
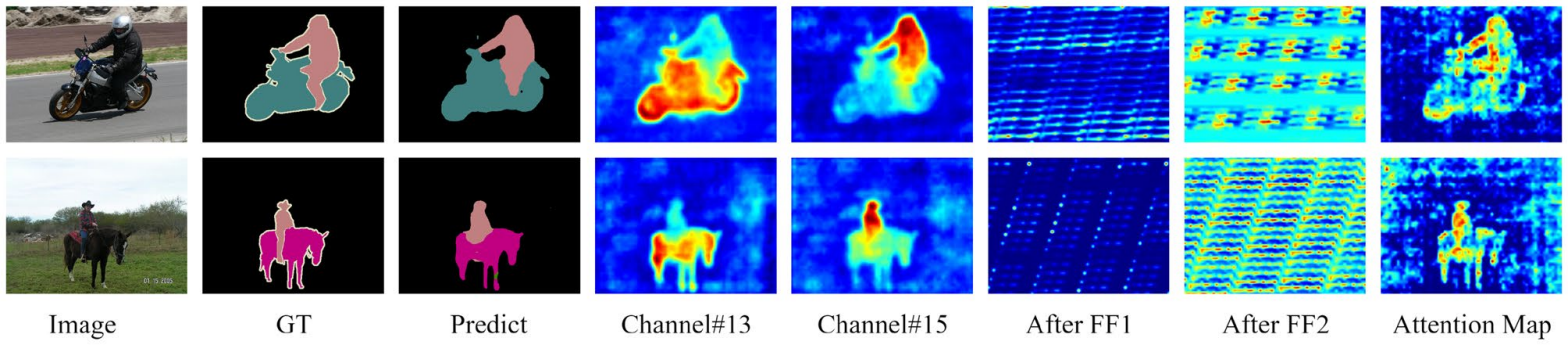
Visualization of PASCAL VOC 2012 (val).

In Fig. 3, we visualize feature maps (come from ResNet50) at different positions. the images in the 4th and 5th columns are the output of the 13th and 15th channels respectively. It shows that the proposed method can get better features. The 6th and 7th columns are the output of the first FF module and the second FF module respectively. After the second FF module, the relationship between each pixel will be calculated, and important information will be given higher weight (such as the bright spot in Fig. 3). The attention map (come from attention matrix), which is generated after aggregating context information, is shown in the final image. It is not difficult to find that the attention map can make the network pay more attention to the area of interest.

### Results on PASCAL VOC 2012

The segmentation results of PASCAL VOC 2012 are show in Table 2. Obviously, the proposed method is better than other methods. Compared with other attention methods, such as DANet and CCNet, the proposed method achieves a higher mIoU (74.5%). In terms of model complexity, the proposed method parameter is only 279 MB, which is about 1/3 less than the most recent mainstream models, such as SPNet and DRANet.

**Table 2:** Performance comparison of different models in PASCAL VOC 2012 (val).

Method	Publication	Weights	mIoU (%)	PA (%)
FCN8s^1^	CVPR2015	180 MB	64.4	90.8
PSPNet^17^	CVPR2017	392 MB	70.8	92
Deeplab^16^	ECCV2018	309 MB	71.8	92.3
UperNet^8^	ECCV2018	817 MB	69.4	91.8
CCNet^28^	ICCV2019	363 MB	71	92.5
DANet^29^	CVPR2019	363 MB	73.2	93
DRANet^30^	IEEE	400 MB	73.8	93.8
SPNet^14^	CVPR2020	346 MB	69.2	91.4
Ours	**–**	279 MB	74.5	93.5

The segmentation results of each category of PASCAL VOC 2012 (val) are shown in Table 3. For categories with a small number and a small area, such as “bicycles” and “bottles”, the proposed model considers rich context information, which make segmentation more delicate and better segmentation results.

**Table 3:** Per-class results on PASCAL VOC 2012 (val).

Method	Background	Airplane	Bicycle	Bird	Boat	Bottle	Bus	Car	Cat	Chair	Cow	Dining	Dog	Horse	Motorcycle	Person	Potted plants	Sheep	Sofa	Train	TV
FCN8^1^	90.6	80.1	55.4	77.7	59.7	57.5	73.5	75.5	77.4	22.7	68	40.5	71.7	67.1	72.6	80.6	48.1	67.5	35.8	71.9	59.4
PSPNet^17^	91.8	86.2	53.8	86.3	65.6	74.1	83.4	81	86.1	27.3	83.1	48.7	78.5	79	82	80.33	46.3	83.9	42.6	74.6	63.5
Deeplab^16^	91.6	88	51.9	84.4	66.3	61.1	87.2	81.8	88.6	30.7	81	59	81.7	74.2	74.7	82.9	51.5	69.5	47.2	84.3	69.3
UPerNet^8^	91.3	84.1	55.4	82.1	65.1	65.7	83.6	78.8	82.8	28.1	74.3	54.3	77.6	75.3	78.4	80.7	47.3	74.5	42.3	71.8	64.6
CCNet^28^	91.6	87.2	48.1	84.2	67.1	74.9	84.8	82.1	87.7	28.9	81.5	44.3	77.6	77.7	71.6	82.3	48.4	82.8	40	81.2	66.9
DANet^29^	92.6	87.3	54.8	87.5	66.8	76.9	86.6	87.2	85.6	29.4	82	56.4	76.5	79.4	79.3	83.8	56.1	78.1	42.3	78.8	69.3
DRANet^30^	92.3	88.6	56.4	85.8	71.7	76	86	85.1	90.6	31.2	80.1	58.5	83.1	74.7	80.2	82.7	55.1	81.8	41.9	82.6	66.3
SPNet^14^	90.6	85.4	51.6	81.7	65.9	71.6	90.7	82.5	85	24.4	76	49.8	77.5	60	71.4	80.2	38.9	79.4	36.8	83.7	70.3
Ours	92.8	88.6	61.3	87.2	69.3	76.9	86.4	83	89.3	28.3	81.9	51.6	82.2	79	80.4	84.1	60.8	79	46.2	85.4	70.1

### Results on CityScapes

We conduct experiments on the CityScapes. The experimental results are shown in Table 4. It can be found that the proposed method achieves 70.3% mIoU, which surpasses the previous mainstream methods. Compared with DANet and DRANet, which also use the self-attention mechanism, the proposed method has 2.9% and 1.1% improvements in mIoU, respectively.

**Table 4:** Segmentation results on the CityScapes (val).

Method	mIoU (%)	PA (%)
FCN8s^1^	62.9	94.4
U-Net^20^	61.3	94.2
PSPNet^17^	67.1	95.2
DeepLab^16^	68.6	95.5
CCNet^28^	66	95
DANet^29^	67.4	95.1
DRANet^30^	69.2	95.7
SPNet^14^	67.6	95.1
Ours	70.3	95.7

**Figure 4:**
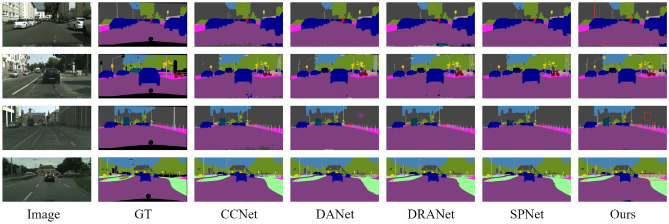
Visualization of CityScapes (val).

As shown in Fig. 4, we visualize the most recent mainstream methods on the CityScapes. The proposed network can obtain a global perspective and accurately segment the image based on contextual information. For example, red boxes for the "road " or "building" in the image, the proposed method can correctly judge the target around the "road " according to the context information and make the segmentation more accurate.

### Results on DLRSD

**Figure 5:**
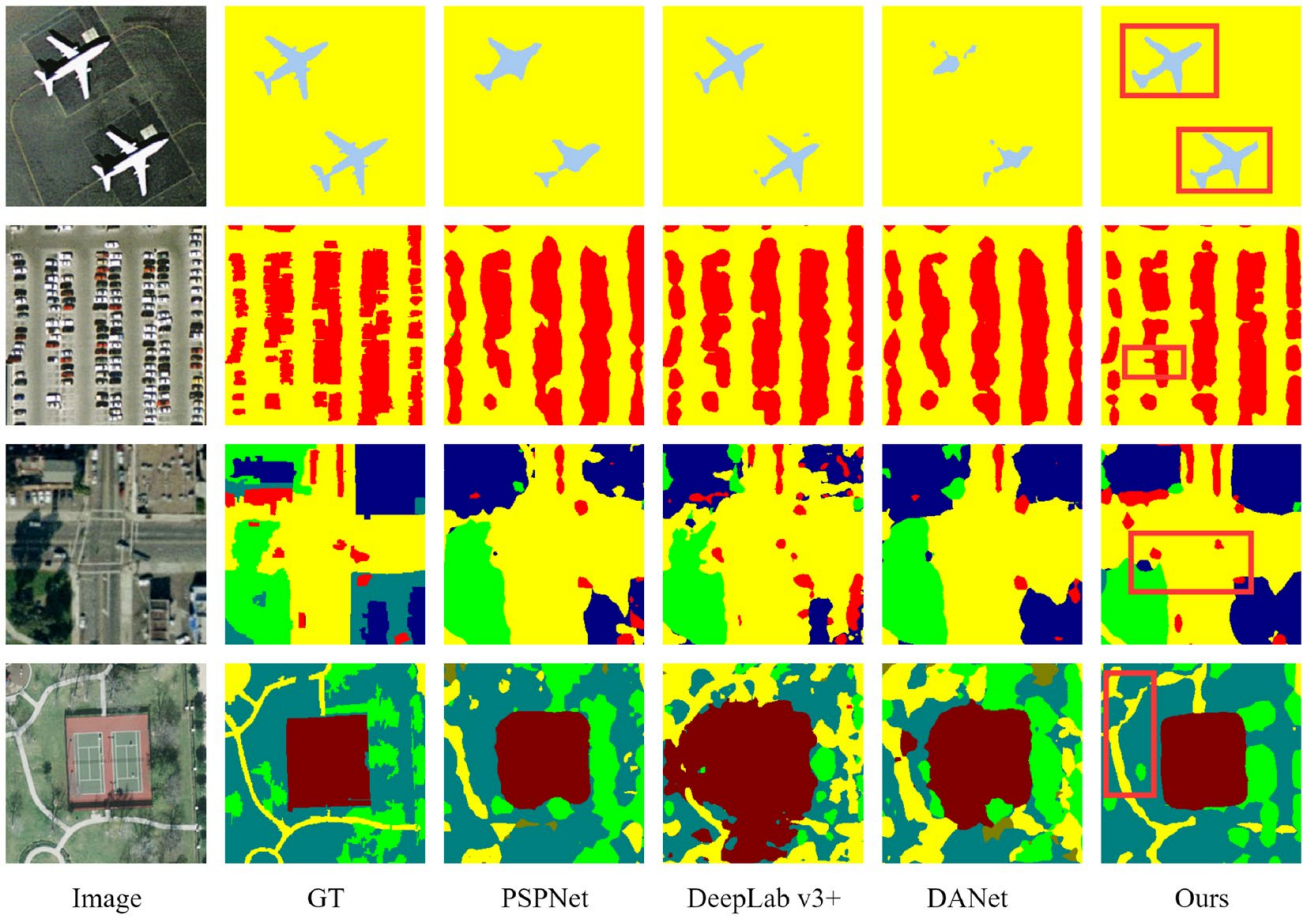
DLRSD (val) dataset visualization results.

The DLRSD dataset is taken from the sky. The background of the objects in the image is complex and the scale is changed drastically, which makes segmentation very difficult. Table 5 shows the verification results on the DLRSD, where FLOPs are measured when the input size is 3 × 248 × 248 and the number of outputs is 17. Compared with DANet, which also uses the self-attention mechanism, the parameter amount of the proposed method is 20% lower than it. Computational complexity can be measured in FLOPs. The proposed network has far fewer FLOPs than the dual-channel self-attention network DANet. Compared with lightweight network LedNet, the proposed method has higher computational complexity, but more computation brings higher segmentation accuracy. The proposed method can automatically aggregate contextual information and achieve 63.9% in the mIoU. Figure 5 shows the corresponding visualization results. In red boxes, for large-scale targets, such as "aircraft", the proposed method can make a more complete segmentation. For small-scale targets, such as "cars", the proposed method can perceive their existence from a global perspective, which is less missed than other methods.

**Table 5:** Segmentation results on the DLRSD (val).

Network	mIoU(%)	PA(%)	FLOPs	Params
FCN 8s^1^	52.7	71.8	6.7 × 10^9^	11,853,788
U-Net^20^	59.3	74.4	1.38 × 10^10^	28,957,521
PSPNet^17^	59.9	77.6	4.33 × 10^10^	23,357,160
DeepLab^16^	61	77.4	4.46 × 10^10^	20,237,465
UperNet^8^	60.3	77.6	1.11 × 10^11^	53,619,560
DANet^29^	59.1	77.2	4.81 × 10^10^	47,565,905
LedNet^12^	56.6	75.7	1.26 × 10^10^	11,419,797
SPNet^14^	55.2	74.6	3.78 × 10^10^	45,371,537
Ours	63.9	79	3.81 × 10^10^	37,862,993

## Conclusion

We propose an efficient self-attention segmentation network (FFANet). FF module that can efficiently capture contextual information is designed. It uses strip pooling to reduce the complexity of the affinity matrix. The spatial transformation is performed through the linear layer to compensate for the information ambiguity caused by the strip pooling. Experiments show that the proposed method can effectively solve long-term dependence and make the segmentation result more accurate. It achieves 74.5% mIoU on the PASCAL VOC 2012, 70.3% mIoU on the CityScapes, and 63.9% mIoU on the DLRSD. Although the use of a linear layer can reduce the information loss caused by the pooling operation, some information will still be lost. Therefore, in the future research, we will explore other feature compression methods to capture global information more effectively (Suppl. Information).

## Supplementary Information

Supplementary Information.

## Data Availability

The PASCAL VOC 2012 dataset can be downloaded from the following link: http://host.robots.ox.ac.uk/pascal/VOC/. The CistyScapes dataset can be downloaded from the following link: https://www.cityscapes-dataset.com/. The DLRSD dataset can be downloaded from the following link: https://sites.google.com/view/zhouwx/dataset#h.p_hQS2jYeaFpV0. The code of the manuscript will be uploaded on the following link: https://github.com/Qianyu1998/FFANet.
