# Supplementary material for: Feature fusion network based on strip pooling
Source: Sci Rep. 2021 Oct 28;11:21270. doi: 10.1038/s41598-021-00585-z (PMC8553855; doi:10.1038/s41598-021-00585-z)
Supplement: Supplementary file 1 — Supplementary Information. [file 41598_2021_585_MOESM1_ESM.docx]

Supplementary Information

Title: Feature Fusion Network Based on Strip Pooling

Author List:

**
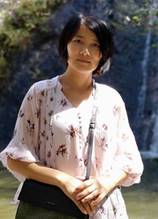
WANG Gaihua** is an associate professor in the School of Electrical and Electronic Engineering, Hubei University of Technology. He is responsible for his work and has a strong sense of innovation. She has made some achievements in teaching and scientific research. She has presided over one project of National Natural Science Foundation of China, two projects of Hubei Provincial Education Department (all of which have been concluded), and participated in a number of national projects. She has published more than 10 papers in EI and above journals, including 2 SCI articles. In the teaching work, he has won many awards such as Outstanding Instructor, Class Tutor Model, and Outstanding Annual Assessment.She guides graduate students to participate in provincial competitions and undergraduate students to carry out undergraduate innovation and entrepreneurship projects.

**
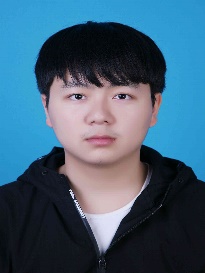
**

**Qianyu Zhai** received the B.E. degree in Hubei University of Technology Engineering and Technology College, Wuhan, China in 2020. He is currently pursuing the M.S. degree with Hubei University of Technology, Wuhan, China.

His current research interests include deep learning and image segmentation.
